# Zinc in Health and Disease Condition—2nd Edition

**DOI:** 10.3390/biom15050609

**Published:** 2025-04-23

**Authors:** Xiang-Ping Chu, Qian Jiang, Yuyang Chu

**Affiliations:** 1 Department of Biomedical Sciences, School of Medicine, University of Missouri-Kansas City, Kansas City, MO 64108, USA; qjiang@umkc.edu; 2 Department of Orthopedic Surgery, Detroit Medical Center, School of Medicine, Wayne State University, Detroit, MI 48201, USA; ychu2@dmc.org

Zinc is an essential trace element that plays a pivotal role in numerous physiological processes, including immune function, antioxidant defense, cellular signaling, and growth [1,2]. It serves as both a structural and catalytic component in over 300 enzymes and modulates the activity of many proteins [3]. Zinc homeostasis is tightly regulated, as both deficiency and excess can lead to a variety of health issues [4]. Emerging research continues to uncover zinc’s involvement in chronic diseases, immune dysregulation, neurological disorders, and its potential therapeutic applications [2,4]. A deeper understanding of zinc’s role in health and disease is therefore critical for advancing both nutritional science and clinical practice.

This Special Issue of “Zinc in Health and Disease Condition—2nd edition” features six papers—four reviews (contributions 1 to 4) and two original research articles (contributions 5 and 6)—that collectively explore the diverse roles of zinc signaling in both physiological and pathological contexts.

*Cirovic* et al. (contribution 1) examine the intricate interplay between zinc and cadmium, a toxic environmental pollutant. While zinc is vital for numerous cellular functions, cadmium mimics zinc and hijacks its transport systems to enter the body. This review highlights how dietary factors and metal transporters—particularly zinc transporters and ZIP families—influence zinc homeostasis and how cadmium disrupts this balance, leading to toxicity. It also underscores the potential of zinc supplementation to mitigate cadmium-induced risks, such as vision loss and bone deterioration, and discusses challenges in defining safe cadmium exposure levels relative to dietary zinc intake.

*Padoan* et al. (contribution 2) provide a comprehensive overview of zinc’s crucial role in pediatric health. Zinc supports immune function, regulates inflammation and oxidative stress, and aids in growth and development. Through modulation of NF-κB and support of lymphocyte activity and cytokine balance, zinc shows therapeutic promise in pediatric conditions, including respiratory, renal, and gastrointestinal diseases. The review emphasizes the relevance of zinc supplementation, even in developed countries, where deficiencies may still impact child health.

*Kawahara* et al. (contribution 3) explore the complex interactions between zinc, copper, and calcium in the brain. These metals are released during neuronal activity and play essential roles in memory and synaptic regulation. Under pathological conditions, such as ischemia, excess zinc can trigger neurotoxicity. The review highlights how copper may exacerbate zinc-induced neuronal damage by disturbing calcium signaling, contributing to the development of vascular-type senile dementia. This “metal triangle” is proposed as a key driver in neurodegenerative processes.

*Maywald and Rink* (contribution 4) discuss the growing prevalence of allergic diseases and the emerging role of zinc in their development and management. Zinc deficiency is linked to immune dysregulation, promoting a Th2-skewed, allergy-prone immune profile. Although clinical trial results vary, evidence suggests that zinc supplementation may alleviate symptoms of conditions such as asthma, allergic rhinitis, atopic dermatitis, and food allergies. The review emphasizes zinc’s complex immunomodulatory functions and its therapeutic potential in allergy management.

*Bauer* et al. (contribution 5) investigate zinc’s protective effects against respiratory damage caused by organic dust exposure in swine production facilities. Using ZinPro™, a highly bioavailable zinc supplement, they show that zinc pre-treatment restores cilia beat frequency in tracheal epithelial cells exposed to swine barn dust. ZinPro effectively prevents PKCε pathway activation without toxicity, suggesting its potential to protect agricultural workers from dust-induced respiratory dysfunction.

*Jiang* et al. (contribution 6) examine how cyanide enhances acid-sensing ion channel (ASIC) activity in cortical neurons. They find that cyanide rapidly increases ASIC currents through a zinc-related mechanism involving the ASIC1a subunit. Mutation of lysine 133 (K133)—a zinc-binding site—abolished the cyanide effect, highlighting zinc’s role in modulating cyanide neurotoxicity. These findings provide novel insights into how environmental toxins interact with zinc signaling in the brain.

Together, these contributions deepen our understanding of zinc’s multifaceted biological roles and underscore its potential as a therapeutic target in diverse health contexts—from environmental toxicology and pediatric care to allergy management and neurodegeneration.

## Abbreviations

The following abbreviations are used in this manuscript:

ASIC	acid-sensing ion channel
PKC	protein kinase C
